# Distinct Activities of Exonuclease 1 and Flap Endonuclease 1 at Telomeric G4 DNA

**DOI:** 10.1371/journal.pone.0008908

**Published:** 2010-01-26

**Authors:** Aarthy C. Vallur, Nancy Maizels

**Affiliations:** 1 Department of Immunology, University of Washington School of Medicine, Seattle, Washington, United States of America; 2 Department of Biochemistry, University of Washington School of Medicine, Seattle, Washington, United States of America; National Cancer Institute, United States of America

## Abstract

**Background:**

Exonuclease 1 (EXO1) and Flap endonuclease 1 (FEN1) are members of the RAD2 family of structure-specific nucleases. Genetic analysis has identified roles for EXO1 and FEN1 in replication, recombination, DNA repair and maintenance of telomeres. Telomeres are composed of G-rich repeats that readily form G4 DNA. We recently showed that human EXO1 and FEN1 exhibit distinct activities on G4 DNA substrates representative of intermediates in immunoglobulin class switch recombination.

**Methodology/Principal Findings:**

We have now compared activities of these enzymes on telomeric substrates bearing G4 DNA, identifying non-overlapping functions that provide mechanistic insight into the distinct telomeric phenotypes caused by their deficiencies. We show that hFEN1 but not hEXO1 cleaves substrates bearing telomeric G4 DNA 5′-flaps, consistent with the requirement for FEN1 in telomeric lagging strand replication. Both hEXO1 and hFEN1 are active on substrates bearing telomeric G4 DNA tails, resembling uncapped telomeres. Notably, hEXO1 but not hFEN1 is active on transcribed telomeric G-loops.

**Conclusion/Significance:**

Our results suggest that EXO1 may act at transcription-induced telomeric structures to promote telomere recombination while FEN1 has a dominant role in lagging strand replication at telomeres. Both enzymes can create ssDNA at uncapped telomere ends thereby contributing to recombination.

## Introduction

Exonuclease 1 (EXO1) and Flap endonuclease 1 (FEN1) belong to the RAD2 family of structure-specific nucleases. They share a core nuclease domain that is remarkably conserved from yeast to mammals [1], and display both 5′-3′ exonuclease activity and 5′ flap endonuclease activity *in vitro* [2], [3]. EXO1 has roles in mismatch repair and recombination [4]; and FEN1 functions in Okazaki fragment maturation, maintenance of simple repeats and prevention of strand slippage [5]. *FEN1* but not *EXO1* is critical in telomeric lagging strand DNA synthesis [6]–[8], while both *EXO1* and *FEN1* contribute to recombination-dependent telomere maintenance in yeast and human cells [9]–[14]. Ablation of *Fen1* but not *Exo1* is lethal in mice [15], [16]. *S. cerevisia*e tolerates ablation of either Exo1 or Rad27 (the *FEN1* homolog), but ablation of both is lethal [17]. Overexpression of Exo1 will partially rescue some deficiencies of *rad27* strains [18], suggesting that these two factors share some overlapping functions.

Telomeres in nearly all eukaryotes are composed of G-rich repeats; in mammals, the telomeric repeat is TTAGGG. G-rich telomeric repeats readily form G4 DNA, a four-stranded structure stabilized by G-quartets, planar arrays of four guanines [19], [20]. G4 DNA structures are implicated as targets in telomere maintenance, and helicases that unwind G4 DNA are closely associated with telomere stability. These include *S. cerevisiae* Sgs1 and human WRN and BLM, members of the RecQ family, which unwind G4 DNA with 3′-5′ polarity, recognizing the G4 structure through their highly conserved RQC domain [21]–[25]; and XPD-family helicases such as *S. cerevisiae* Srs2, nematode DOG-1 and mammalian FANCJ and RTEL1, which unwind DNA with 5′-3′ polarity [26]–[29]. Deficiency in RTEL1, which is functionally equivalent to Srs2 [30], results in a telomeric fragility phenotype similar to that caused by deficiency of BLM [31]. WRN helicase is essential for efficient replication of telomere lagging strands [32]; and BLM helicase represses telomere fragility [31] and associates with telomeres in telomerase-deficient cells [33]. FEN1 and EXO1 interact both physically and functionally with WRN and BLM helicases [34]–[38], which may promote function in telomere maintenance.

Like the telomeres, the immunoglobulin (Ig) heavy chain switch regions are composed of G-rich repeats with considerable potential to form G4 DNA. The Ig switch regions are targets for class switch recombination, a process of DNA deletion that enables a B cell to juxtapose a new constant region to the expressed variable region [39], [40]. EXO1 is necessary for efficient class switch recombination [41], and we recently showed that human EXO1 (hEXO1) and human FEN1 (hFEN1) exhibit distinct activities on substrates representing switch recombination intermediates [42]. Ig class switch recombination is induced by transcription; and hEXO1 but not hFEN1 excised characteristic G-loop structures formed by transcribed switch region substrates [43]. Those experiments identified a property of EXO1 that could contribute to its function not only at the Ig switch regions, but also at other transcribed genomic domains with potential to form G4 DNA.

Those results prompted us to examine the biochemical activities of EXO1 and FEN1 that might contribute to telomere maintenance or instability, using substrates that recapitulate structures predicted to form upon telomere replication, uncapping, recombination or transcription. We show that hFEN1 but not hEXO1 cleaves 5′ flaps containing telomeric G4 DNA, or other G4 DNA. This is consistent with the importance of FEN1 and not EXO1 in telomere lagging strand replication, but provides a surprising exception to the documented inactivity of FEN1 at other structured 5′ flaps *in vitro* [44], [45]. We demonstrate that hEXO1 and hFEN1 both exhibit robust exonucleolytic activity on both strands of substrates with G-rich 3′ telomeric tails, enabling either enzyme to expose a single-stranded region for recombination, or to participate in removal or formation of a G-rich tail. We show that hEXO1 but not hFEN1 excises G-loops formed at transcribed telomeric repeats, suggesting a mechanism by which telomeric transcription could promote recombination to maintain telomere length in the absence of telomerase. These results define new activities for these enzymes, and provide mechanistic understanding of how deficiencies in these enzymes cause distinct telomeric phenotypes.

## Results

### hFEN1, but Not hEXO1, Cleaves Telomeric 5′ G4 Flaps, Possible Intermediates in Lagging Strand Replication

**Figure 1:**
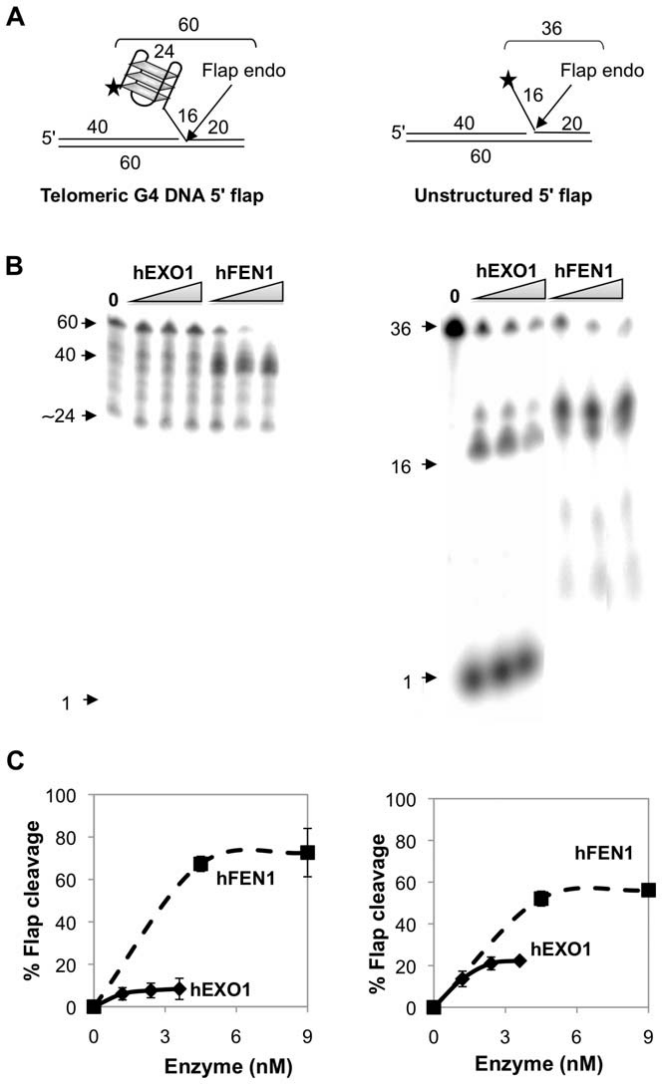
hFEN1, but not hEXO1, cleaves 5′ telomeric G4 DNA. (A) Diagram of substrates bearing a G4 or unstructured 5′ flaps. Lengths of oligonucleotides, flaps and duplex regions are indicated; asterisks denotes 5′ end-label. (B) Products of digestion of 5′ flap substrates shown in Panel A by hEXO1 (0, 1.2, 2.4 and 3.6 nM) and hFEN1 (0, 4.5, 9.0 and 18 nM). Arrows indicate undigested (60 nt or 36 nt) 5′-labeled DNA substrate, products of flap endonuclease digestion, and 1 nt product of exonuclease digestion. Heterogenous flap cleavage products like those evident here are characteristic of hFEN1 activity [65]. (C) Quantitation of flap cleavage activity of hEXO1 (0, 1.2, 2.4 and 3.6 nM; diamonds) and hFEN1 (0, 4.5, 9 and 18 nM; squares) on substrates shown in Panel A.

The canonical activity of FEN1 is endonucleolytic excision of a 5′ flap from a duplex substrate, which represents the intermediate in processing Okazaki fragments during lagging strand replication [5]. During telomeric lagging strand replication, a 5′ flap may become structured as G4 DNA. A variety of 5′ flap structures have been shown to inhibit FEN1 activity *in vitro* [45]. This raised the question of whether the presence of G4 DNA at a 5′ flap would affect cleavage by EXO1 or FEN1. To address this, we assayed cleavage of two duplex substrates bearing 5′ flaps, one carrying telomeric G4 DNA, the other unstructured (Fig. 1A). hFEN1 displayed robust flap endonuclease activity on the substrate bearing a 5′ telomeric G4 DNA flap (70% cleavage at 4.5 nM enzyme), while hEXO1 was inactive on this substrate (Fig. 1B, C, left). Both enzymes were active on the substrate bearing an unstructured 5′ flap, although hFEN1 was somewhat more active than hEXO1. hFEN1 activity at a 5′ G4 DNA flap was comparable to its activity on an unstructured 5′ flap (Fig. 1B, C, right). Thus, unlike other structures, 5′ G4 DNA does not inhibit hFEN1 flap endonuclease activity; but 5′ G4 DNA does inhibit hEXO1 flap endonuclease activity.

### 3′ G4 Telomeric Tails Inhibit Flap Cleavage by hEXO1 but Not hFEN1

**Figure 2:**
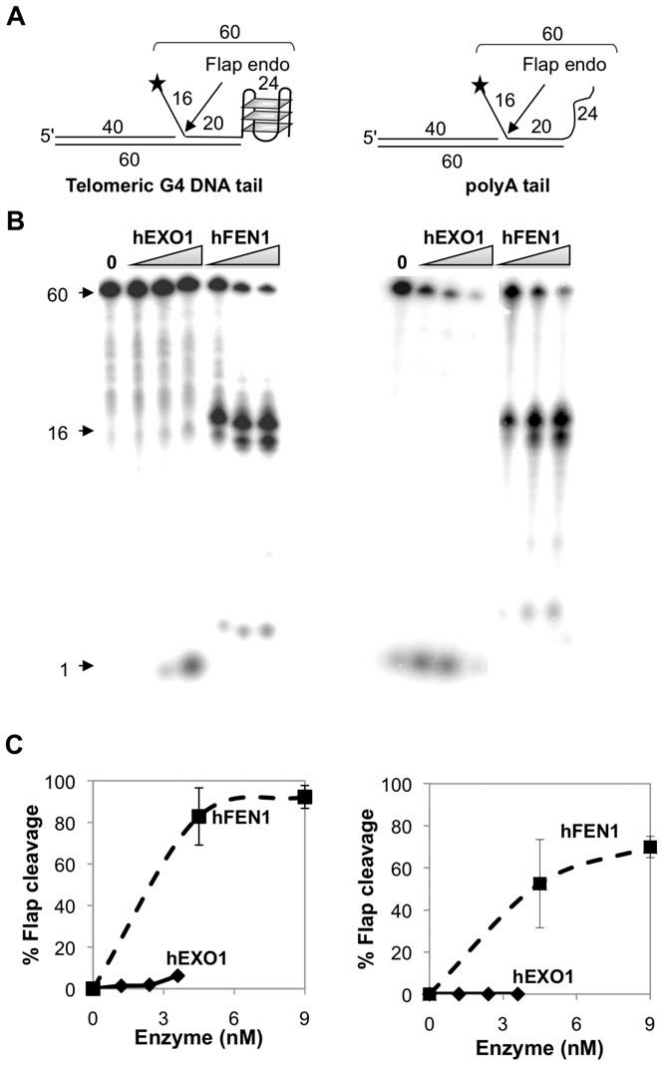
A 3′ G4 DNA tail inhibits flap endonuclease activity of hEXO1 but not hFEN1. (A) Diagram of substrates bearing a 5′ flap and 3′ telomeric G4 DNA or polyA tails. Lengths of oligonucleotides, flaps, duplex regions and 3′ tails are indicated; asterisks denote 5′ end-label. (B) Products of digestion of substrates shown in Panel A by hEXO1 (0, 1.2, 2.4 and 3.6 nM) and hFEN1 (0, 4.5, 9.0 and 18 nM). Arrows indicate 60 nt 5′-labeled DNA substrate, 16 nt flap cleavage product, and 1 nt exonucleolytic cleavage product. (C) Quantitation of flap cleavage activity of hEXO1 (0, 0.6, 1.2 and 2.4 nM; diamonds) and hFEN1 (0, 4.5, 9 and 18 nM; squares) on substrates shown in Panel A.

The novel activity of FEN1 at a 5′ G4 DNA flap prompted us to test activities of hEXO1 or hFEN1 at unstructured 5′ flaps on substrates bearing either a 3′ telomeric G4 DNA tail or an unstructured tail (Fig. 2A). hEXO1 exhibited essentially no flap endonucleolytic activity and very modest exonucleolytic activity on the G4 DNA substrate (Fig. 2B, C, left). This was somewhat surprising, because hEXO1 typically exhibits both endonuclease and exonuclease activities at 5′ flaps [42], [46]. Thus, a 3′ tail may inhibit activity of hEXO1 at a 5′ flap. In contrast, hFEN1 exhibited very robust flap endonuclease activity on the substrate with a G4 DNA tail (more than 80% of this substrate cleaved at 4.5 nM hFEN1; Fig. 2B, C, left). hFEN1 was somewhat less active on the substrate with an unstructured 3′ tail (60% cleavage at 4.5 nM hFEN1; Fig. 2B, C, right), but was comparably active on this substrate as on a substrate carrying no tail (Figs. 1C and 2C). These results show that a 3′ tail can inhibit hEXO1, but not hFEN1; and suggest that a 3′ G4 DNA tail may even stimulate hFEN1.

### Nontelomeric and Telomeric 5′ or 3′ G4 DNA Flaps Comparably Affect hEXO1 and hFEN1 Flap Endonuclease Activities

**Figure 3:**
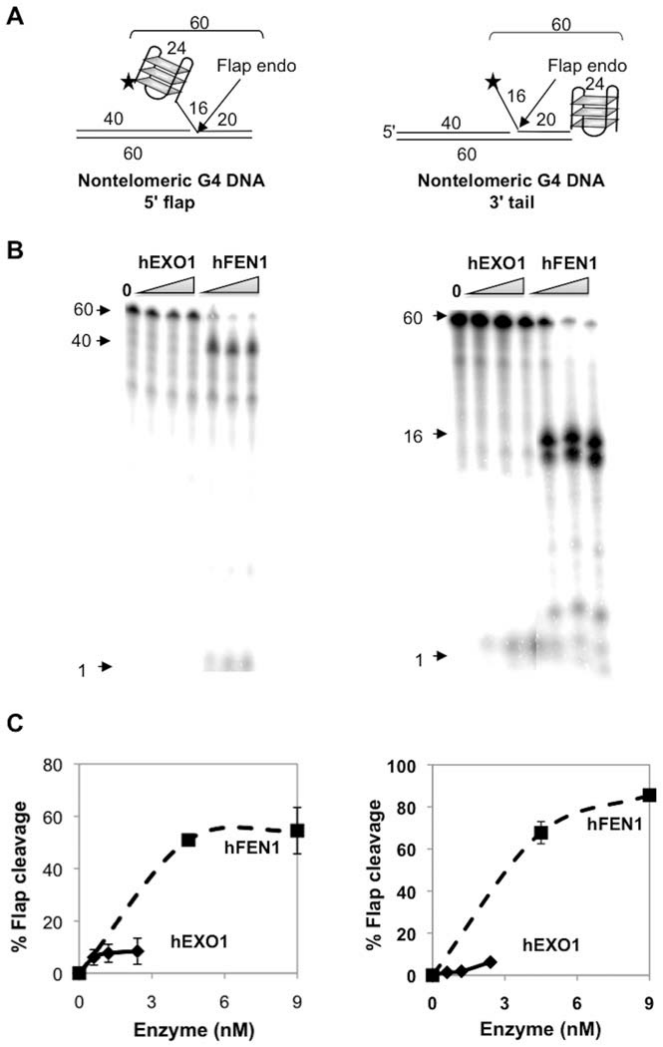
hFEN1, but not hEXO1, cleaves 5′ nontelomeric G4 DNA flaps and 5′ flaps adjacent to 3′ nontelomeric G4 DNA tails. (A) Diagram of substrates bearing 5′ or 3′ nontelomeric G4 DNA tails. Lengths of oligonucleotides, flaps, duplex regions and 3′ tails are indicated; asterisk denotes 5′ end-label. (B) Products of digestion of substrates shown in Panel A by hEXO1 (0, 1.2, 2.4 and 3.6 nM) and hFEN1 (4.5, 9.0 and 18.0 nM). Arrows indicate 60 nt undigested 5′-labeled DNA substrates, products of flap endonuclease digestion, and 1 nt product of exonuclease digestion. (C) Quantitation of flap cleavage activity of hEXO1 (0, 0.6, 1.2 and 2.4 nM; diamonds) and hFEN1 (0, 4.5, and 9.0 nM; squares) on substrates shown in Panel A.

The results in Figs. 1 show that telomeric G4 DNA inhibits the flap endonuclease activity of hEXO1 but not hFEN1. To ask if this is specific for telomeric sequences, we assayed substrates of similar structures but bearing G4 DNA of identical length but formed from the synthetic, nontelomeric sequence, CCTGGGCTAGGGATCGGGACCGGG, within the 5′-flap or 3′ tail (Fig. 3A). hFEN1 was very active on both substrates, and hEXO1 was inactive (Fig. 3B, C). Thus, the effect of G4 structures on the flap endonuclease activities of these enzymes is general, and not specific to telomeric G4 DNA.

### hEXO1 and hFEN1 Excise from a Nick on a Strand Bearing a 3′-G4 DNA Telomeric Tail

**Figure 4:**
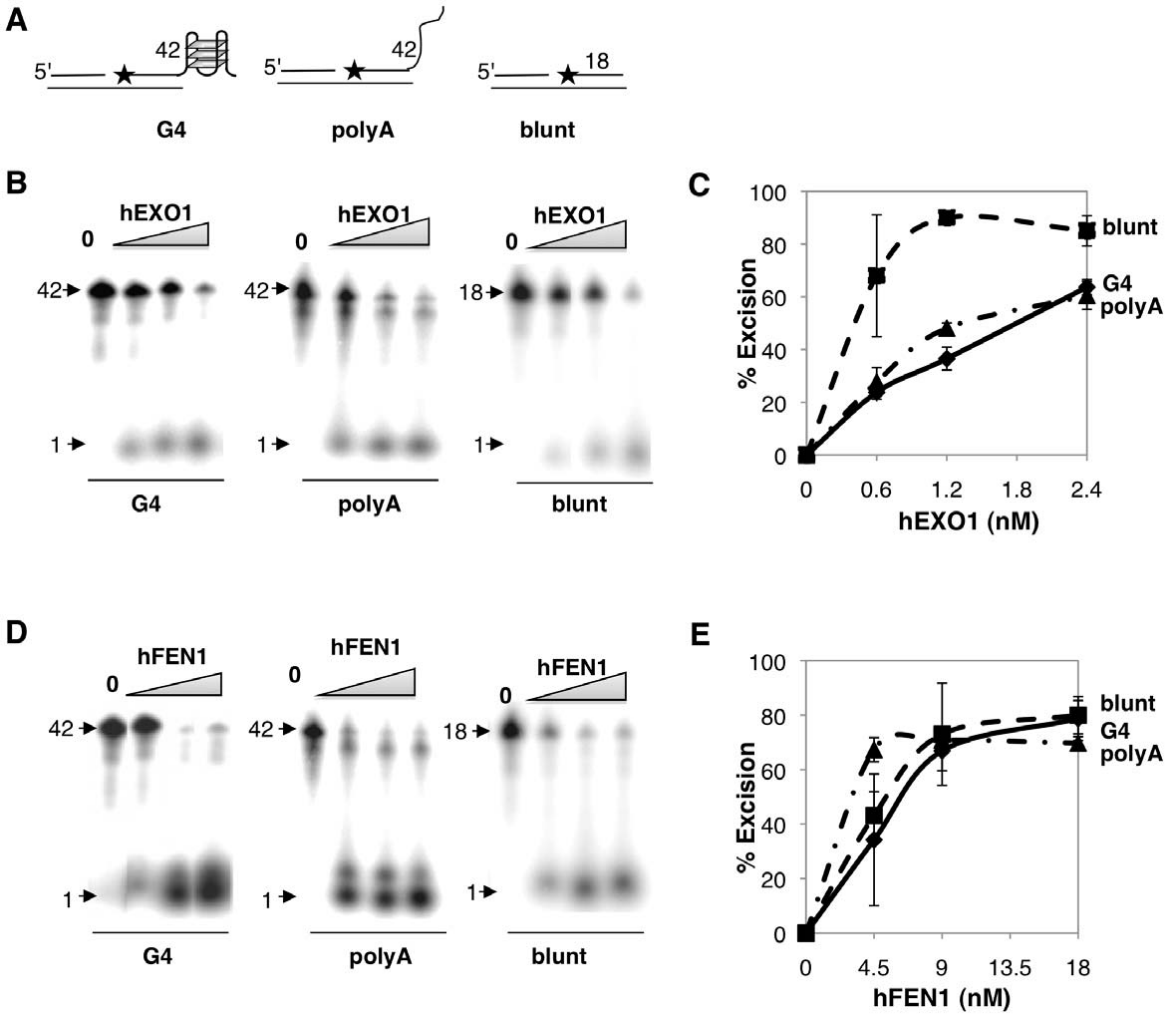
hEXO1 and hFEN1 excise from a nick on a strand bearing a 3′ telomeric tail. (A) Diagram of duplex substrates bearing an internal nick adjacent to a 5′ end-labeled 3′-G4 DNA (TTAGGG)_4_ tail, 3′ poly(A)_24_ tail, or blunt end. The length of the labeled oligonucleotide is indicated; asterisks, end-label. (B) Products of digestion of each substrate by hEXO1 (0, 0.6, 1.2 and 2.4 nM). Arrows indicate 42 or 18 nt undigested 5′-lableled DNA substrate and 1 nt excision product. (C) Quantitation of hEXO1 excision of blunt-ended duplex substrates (squares), substrates bearing 3′ G4 DNA overhangs (diamonds) and substrates bearing poly(A)_24_ tails (triangles). (D) Products of digestion by hFEN1 (0, 4.5, 9.0 and 18 nM). Notations as in panel B. (E) Quantitation of hFEN1 excision of blunt-ended duplex substrates (squares), substrates bearing 3′ G4 DNA overhangs (diamonds) and substrates bearing polyA tails (triangles).

Both EXO1 and FEN1 can excise from nicks in blunt-ended duplex substrates [42], [47], but whether a G4 DNA tail affects excision has not been tested. To do so, we assayed activities of these two enzymes on a nicked DNA duplex bearing a 24 nt 3′ (TTAGGG)_4_ tail (Fig. 4A), mimicking a structure that may form *in vivo* at an uncapped telomeric end. The 3′ tail spontaneously forms intramolecular G4 DNA in our standard assay conditions (Fig. 1A). Both a duplex bearing a 3′-polyA tail and a blunt duplex were included as controls, in order to discriminate between the effects of the G4 structure and a disordered tail. hEXO1 was active on a substrate bearing a G4 tail, but comparably active on a substrate bearing a 3′-polyA tail and slightly more active on a control blunt-ended duplex (Fig. 4B, C). hFEN1 was equally active on all three substrates (Fig. 4D, E). Thus, both enzymes can initiate at a nick to degrade the G-rich strand of DNA carrying a telomeric overhang. This activity could expose the C-rich strand for recombination, but also has the potential to destabilize the telomere by removing the G-rich tail.

### A Telomeric Tail, but Not a polyA Tail, Stimulates hEXO1 and hFEN1 Excision on the Opposite Strand

**Figure 5:**
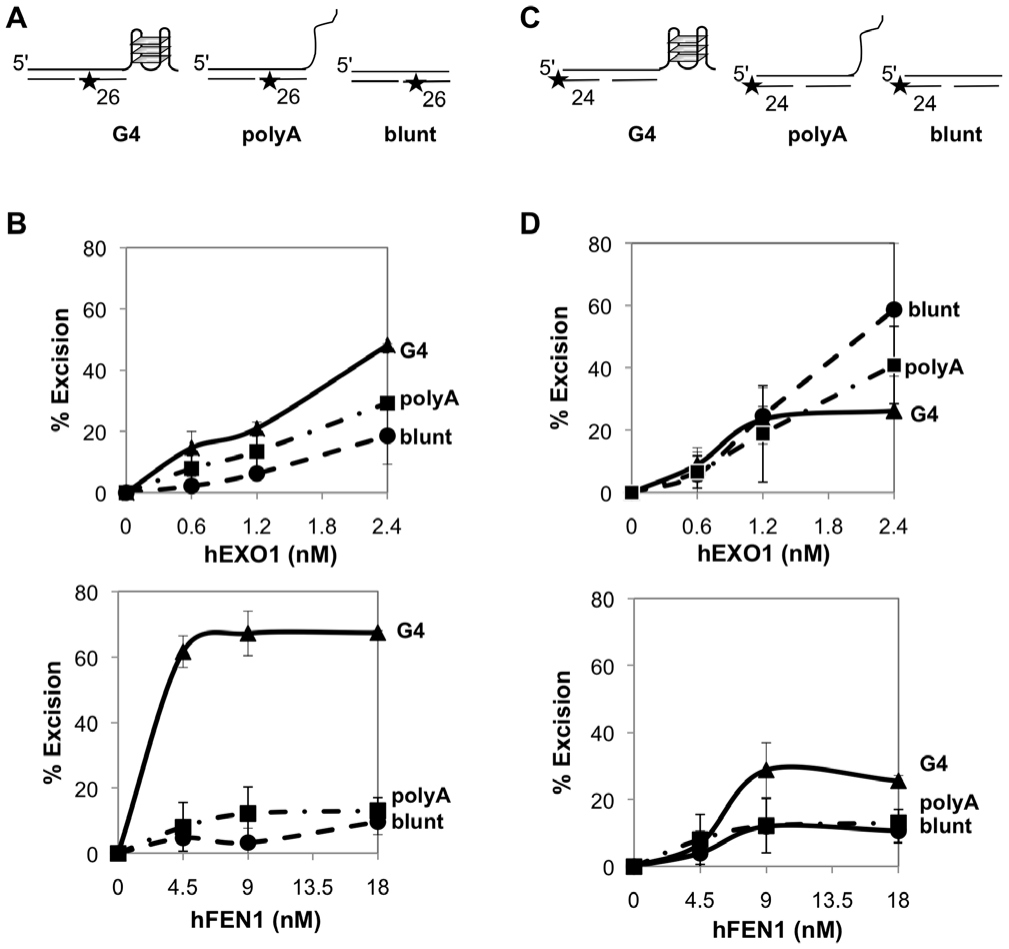
A telomeric tail stimulates hEXO1 and hFEN1 excision on the opposite strand. (A) Diagram of duplex substrates for assay of excision opposite a G4 DNA (TTAGGG)_4_ tail, a polyA_24_ tail, or a blunt end. Sizes of 3′-labeled oligonucleotides are indicated; asterisks, end-label. (B) Quantitation of hEXO1 (0, 0.6, 1.2 and 2.4 nM, above) or hFEN1 (0, 4.5, 9.0 and 18 nM, below) excision of substrates diagrammed in panel A. G4 DNA tails, triangles; 3′ poly(A)_24_ tails, squares and blunt ends, circles. (C) Diagram of duplex substrates for assay of excision at a nick on the strand opposite a G4 DNA (TTAGGG)_4_ tail, a 3′-poly(A)_24_ tail, or a blunt end, and 3′ end-labeled on the nicked strand distal to the tail. Sizes of 3′-labeled oligonucleotides are indicated; asterisks, end-label. (D) Quantitation of hEXO1 (0, 0.6, 1.2 and 2.4 nM, above) or hFEN1 (0, 4.5, 9.0 and 18 nM, below) excision of substrates diagrammed in panel C. Notations as in panel B.

We then compared the ability of hEXO1 or hFEN1 to create single-stranded regions on DNA duplex substrates on the strand opposite a telomeric tail. Substrates were internally labeled by 3′-filling to enable assays of 5′-3′ exonucleolytic digestion initiating opposite the structured end (Fig. 5A). hEXO1 proved to be considerably more active on the substrate bearing a 3′ telomeric tail than on the substrates bearing a polyA tail or a blunt end (Fig. 5B, upper), while hFEN1 displayed robust exonucleolytic activity on the substrate bearing a 3′ telomeric tail, but little activity on the other substrates (Fig. 5B, lower). The activities of hEXO1 and hFEN1 on the substrate bearing a 3′ telomeric tail were comparable: 50% of the substrate was digested by approximately 3 nM enzyme. Thus, a G4 DNA telomeric tail, but not a polyA tail or blunt end, can stimulate 5′-3′ excision by either hEXO1 or hFEN1 on the opposite strand.

We also compared digestion of these same substrates initiated by hEXO1 and hFEN1 at an internal nick (Fig. 5C). hEXO1 was most active on the blunt duplex substrate, but less active on the substrates bearing polyA tail or telomeric tail (Fig. 5D, upper). A telomeric tail appeared to inhibit excision from a nick on the opposite strand on duplex DNA, even though separated from the nick by 26 base pairs. hFEN1 was less active than hEXO1 on these substrates, but was approximately twice as active on the substrate bearing the telomeric tail than on the blunt-ended or polyA-tailed substrate (Fig. 5D, lower).

### Transcribed Telomere Repeats Are Excised by hEXO1 but Not hFEN1

**Figure 6:**
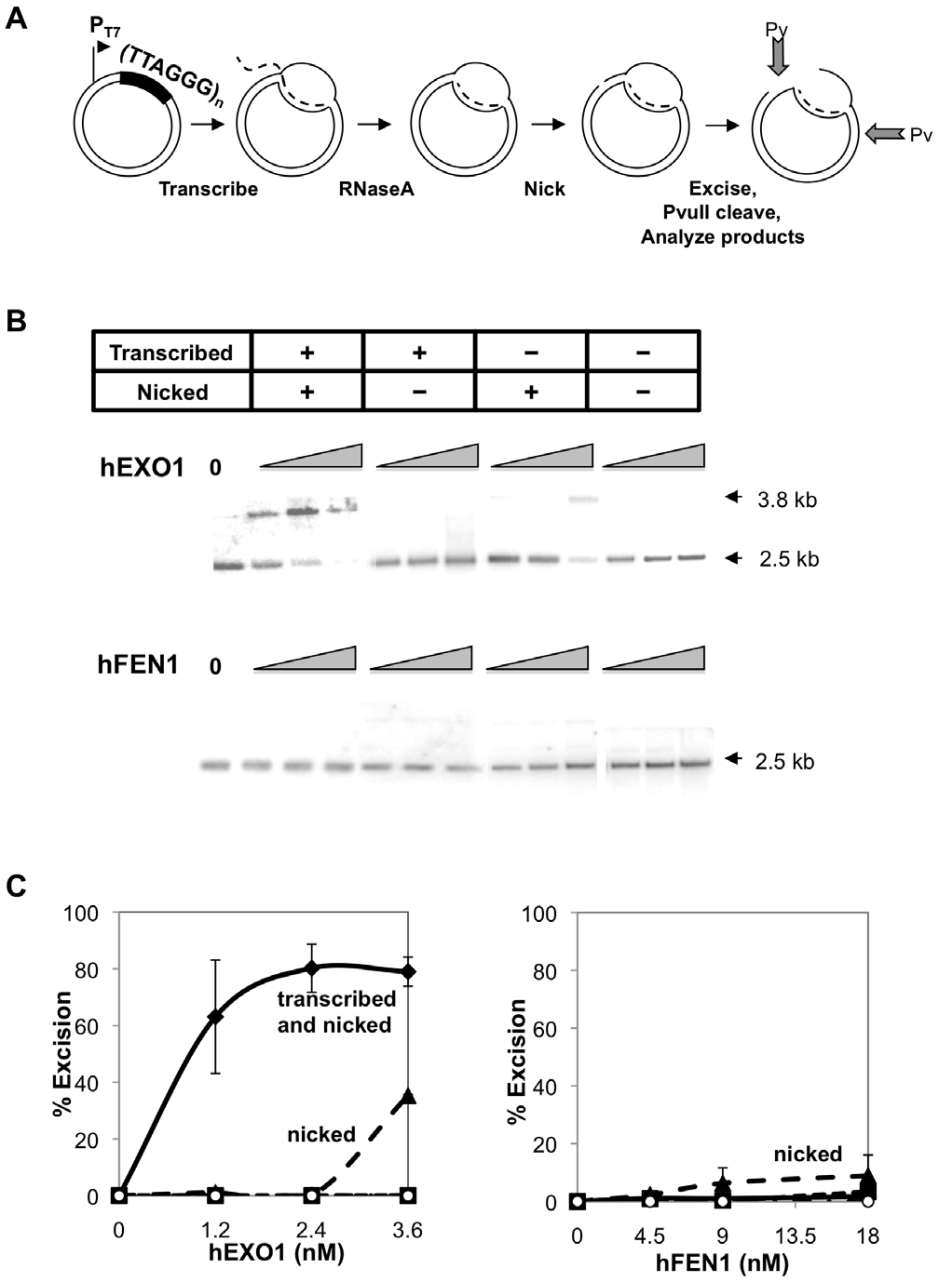
Transcribed telomere repeats are excised by hEXO1 but not hFEN1. (A) pTELN telomeric substrates. T7 promoter (P_T7_); 800 human (TTAGGG) telomeric repeat (dark fill); RNA transcript (dashed line); and PvuII cleavage sites (Pv; arrows) are indicated. (B) Products of PvuII digests of pTELN substrates, which had been transcribed or nicked, as indicated, following digestion with hEXO1 (above, 0.6, 1.2, 2.4 and 4.8 nM) or hFEN1 (below, 0. 4.5, 9 and 18 nM). Fragments were phosphor-imaged and quantitated following transfer and indirect labeling with a probe to the T7 promoter. Arrows denote the 3.8 kb full-length plasmid, and the 2.5 kb PvuII product. (C) Quantitation of products of excision of transcribed, nicked pTELN (diamonds), transcribed, pTELN (squares), nicked pTELN (triangles) and supercoiled pTELN (open circles) by 0, 1.2, 2.4 and 3.6 nM hEXO1 (left) and by 0, 4.5, 9 and 18 nM hFEN1 (right). Open circles and squares are overlapping for hEXO1.

Telomeric repeats are transcribed *in vivo*, and the G-rich strand is the non-template strand [48], [49]. Transcription of telomeres and other G-rich sequences produces characteristic structures, called G-loops, which contain a stable RNA/DNA hybrid on the C-rich template strand, and G4 DNA interspersed with single-stranded regions on the G-rich nontemplate strand [43], [50]. We tested activity of hEXO1 and hFEN1 on G-loops formed by transcribed telomeric sequences formed in the pTELN plasmid, which bears 800 bp of TTAGGG human telomeric repeat downstream of a T7 promoter (Fig. 6A). pTELN was transcribed, free transcript removed by RNaseA treatment, and DNA nicked. We then compared digestion by hEXO1 and hFEN1 of substrates that had been transcribed and nicked substrates, transcribed but not nicked, nicked but not transcribed, and the supercoiled template DNA. Digestion was quantitated by monitoring loss of one of the PvuII sites that occurs as a consequence of excision from the nick, and which results in production of a 3.8 kb PvuII fragment. hEXO1 proved to be quite active on the transcribed, nicked telomeric substrates: approximately 80% of the substrate was excised by 2.4 nM hEXO1 (Fig. 6B, upper; Fig. 6C), comparable to hEXO1 activity at transcribed Ig switch regions [42]. hEXO1 was also somewhat active on the nicked substrate, consistent with its documented activity on nicked DNA in mismatch repair. hEXO1 was not active either on the supercoiled template, or on transcribed, unnicked DNA. In contrast, hFEN1 showed no activity on any of the substrates (Fig. 6B lower; Fig. 6C). These results identify EXO1 as a candidate activity for resection of DNA at transcribed telomeres.

## Discussion

We have shown that hEXO1 and hFEN1 exhibit both shared and distinct biochemical activities on telomeric substrates. These activities correlate with the roles of EXO1 and FEN1 in telomere maintenance, as determined by genetic analysis, and thus provide mechanistic insight into the distinct telomeric phenotypes caused by deficiencies in these enzymes. Notably, distinct activities of each enzyme depended upon a G4 structure, supporting the importance of G4 DNA recognition in telomere maintenance.

### FEN1 May Process G4 Structures Formed during Lagging Strand Replication

hFEN1 but not hEXO1 displayed robust endonuclease activity on substrates bearing telomeric or nontelomeric 5′ G4 DNA flaps, representing intermediates in lagging strand replication. This contrasts with the comparable activities of these enzymes at unstructured 5′ flaps [42]. hFEN1 activity on these structures constitutes an exception to the documented inhibition of FEN1 *in vitro* by other 5′-structures, such as ds DNA and hairpins formed by triplet repeats [44], [45]. Moreover, while either a G4 DNA tail or unstructured tail inhibited endonucleolytic cleavage by hEXO1, the 5′ flap endonuclease activity of hFEN1 was not impaired by proximity of a 3′ tail.

G4 DNA structures may create blocks to replication at telomeres [31]. The ability of FEN1 but not EXO1 to cleave a flap containing G4 DNA would then explain both the instability of telomeric lagging strands that occurs in the absence of FEN1, and the inability of Exo1 overexpression to rescue this instability in *S. cerevisiae* [6]–[8]. In human cells, telomere lagging strand instability is observed in the absence of FEN1 [8], and also in the absence of the RecQ family helicase, WRN [32]. WRN has robust G4 DNA unwinding activity [23], and interacts with and stimulates FEN1 [34], [51], [52]. Like most RecQ family helicases, WRN contains a high affinity G4 DNA binding domain, the RQC domain [53], which could promote FEN1 recognition of G4 DNA *in vivo*. Alternatively, FEN1 may itself specifically recognize G4 structures, a possibility supported both by the robust activity of hFEN1 on 5′ flaps containing G4 DNA, and by the ability of a G4 3′ tail, but not a polyA tail or blunt end, to stimulate hFEN1 exonucleolytic activity.

### FEN1 and EXO1 Activities at Telomeric G4 DNA Tails

Both hEXO1 and hFEN1 were able to excise substrates bearing 3′ telomeric tails, which resemble uncapped telomeres. This activity could expose the C-rich strand for recombination, or remove the G-rich tail and thereby destabilize the telomere. Stabilization of telomeres that occurs upon ablation of *Exo1* in telomerase-deficient mice [54] may be one manifestation of this activity.

hFEN1 and hEXO1 were also very active at the 5′ end of the strand opposite a telomeric tail, where hFEN1 in particular was stimulated by G4 DNA. *In vivo*, excision from this end could lead to an increase in the length of a 3′ telomeric tail, at the expense of duplex DNA. The roles of FEN1 and EXO1 in telomerase-deficient cells have been ascribed to functions in recombination, including exposing single-stranded DNA or resolving structures produced by branch migration. Our results identify another possible function, in exposing G-rich regions for 3′ tail formation.

### Telomere Transcription May Enable EXO1 to Create Substrates for Recombination

*In vivo*, telomeres are transcribed from promoters in subtelomeric regions [49], [55]. Transcription of G-rich telomeric sequences results in formation of characteristic G-loop structures, which contain a stable RNA/DNA hybrid on the C-rich template strand and G4 DNA interspersed with single-stranded regions on the G-rich strand [43]. hEXO1 (but not hFEN1) excised telomeric G-loops, in a reaction that parallels excision of G-loops formed within transcribed Ig switch regions [42]. While the function of telomeric transcription is not yet understood, it has been shown to be more active in cells that lack telomerase and depend upon recombination for telomere maintenance [56]. Our results suggest that one function of telomere transcription may be to promote formation of recombinogenic structures that are substrates for EXO1. Because telomere transcription initiates in subtelomeric regions, promoter-proximal sequences will be enriched among these substrates, conferring the potential to transfer long regions of sequence to the recipient telomere.

### G4 DNA as a Target of FEN1 and EXO1

The results documented here show that both FEN1 and EXO1 must be included on the growing list of critical DNA maintenance and repair factors that are active at G4 DNA. This list also includes BLM, WRN, Sgs1 and related RecQ family helicases [21], [22], [24], [32], [57], [58]; and XPD-family helicases such as FANCJ, RTEL1, DOG-1 and Srs2 [26]–[29]. hFEN1 and hEXO1 activities were stimulated by both telomeric and nontelomeric sequences, so both enzymes could act upon G4 DNA that formed outside the telomeric regions. This may explain why G-rich ribosomal DNA repeats [59] and the G-rich minisatellites CEB1 and MS32 [60], [61] are unstable in *S. cerevisiae rad27* strains which lack the FEN1 homolog. The evidence that robust activity of hFEN1 was evident on structures on which hEXO1 exhibited limited or no activity suggests that EXO1 might not be able to compensate for FEN1 to maintain stability of genomic regions with high potential for G4 DNA formation.

The G4 DNA signature motif, and consequently potential for G4 DNA formation, is unevenly distributed and also selected in the human genome, characterizing not just specialized domains like the telomeres, ribosomal DNA and Ig switch regions, but also promoters and specific functional classes of genes [62]–[64]. The ability of G4 structures to stimulate FEN1 and EXO1 not only identifies these enzymes as key to stability of regions bearing the G4 motif, but also raises the further possibility that the G4 motif may confer special localized properties in replication or recombination.

## Materials and Methods

### DNA Substrates

Oligonucleotides were 5′ end-labeled with γ^32^P-dATP using T4 polynucleotide kinase (NEB) or 3′-labeled by filling with Exo^−^ Klenow polymerase (NEB); and free nucleotides removed using a G50 spin column (GE). Synthetic oligonucleotide substrates (below) were generated by heating oligonucleotides at equimolar concentrations at 90°C in TE containing 40 mM KCl (which promotes G4 DNA formation), followed by slow cooling and overnight incubation at room temperature. Annealed substrates were resolved on an 8% nondenaturing polyacrylamide gel containing 10 mM KCl, eluted, and stored in TE containing 10 mM KCl. Formation of G4 structures was confirmed by analysis of labeled substrates on native gels containing 10 mM KCl, where more than 90% of the label migrated with retarded mobility, diagnostic of G4 DNA (Figure S1).

Substrates bearing a 5′ telomeric or nontelomeric G4 flap were generated by annealing either 5′-labeled 5′-(TTAGGG)_4_ATCATGGCTTGCGATACTTTCCCCGTCTAGTCGCTA-3′ or 5′- CCTGGGCTAGGGATCGGGACCGGGATCATGGCTTGCGATACTTTCCCCGTCTAGTCGCTA-3′ respectively, to 5′-TAGCGACTAGACGGGGAAAGCCGAATTTCTAGAATCGAAAGCTTGCTAGCAATTCGGCGA-3′ and 5′-TCGCCGAATTGCTAGCAAGCTTTCGATTCTAGAAATTCGG-3′. Substrates bearing a 3′ unstructured flap were generated by annealing the latter two oligonucleotides to 5′ end-labeled 5′-ATCATGGCTTGCGATACTTTCCCCGTCTAGTCGCTA-3′. Substrates bearing a 3′ G4 telomeric, polyA or nontelomeric tail were generated by annealing the latter two oligonucleotides to 5′-ATCATGGCTTGCGATACTTTCCCCGTCTAGTCGCTA(TTAGGG)_4_-3′, 5′-ATCATGGCTTGCGATACTTTCCCCGTCTAGTCGCTA(A)_24_-3′ or 5′-ATCATGGCTTGCGATACTTTCCCCGTCTAGTCGCTACCTGGGCTAGGGATCGGGACCGGG-3′, respectively.

Blunt-ended substrates bearing an internal nick were generated by annealing 5′-GTAGAGGATCTAAAAGACTT-3′ and 5′ end-labeled 5′-CGTCCGAAAGTTGCTGAACT-3′ to 5′-AGTTCAGCAACTTTCGGACGAAGTCTTTTAGATCCTCTAC-3′. Substrates bearing an internal nick on the same strand as a telomeric or polyA tail were generated by annealing 5′-TAGCGACTAGACGGGGAAAGTATCGCAAGCCATGAT-3′ to 5′- ATCATGGCTTGCGATACT-3′ and 5′ end-labeled 5′-TTCCCCGTCTAGTCGCTA(TTAGGG)_4_-3′ or 5′-TTCCCCGTCTAGTCGCT(A)_24_-3′, respectively.

Substrates for assaying excision from the 5′ end opposite a 3′ telomeric or polyA tail or blunt end were generated by annealing 5′-GAGGTCACTCCAGTGAATTCGAG-3′ to 5′-GGAAAGTCACGACCTAGACACTGCGAGCTCGAATTCACTGGAGTGACCTC(TTAGGG)_4_-3′, 5′-GGAAAGTCACGACCTAGACACTGCGAGCTCGAATTCACTGGAGTGACCTC(A)_24_-3′, or 5′-GGAAAGTCACGACCTAGACACTGCGAGCTCGAATTCACTGGAGTGACCTC-3′, respectively; 3′-labeling by filling with α-^32^P-dCTP and 0.33 mM cold dTTP; and annealing 5′-GCAGTGTCTAGGTCGTGACTTT-3′.

Substrates for assaying excision from a nick on the strand opposite a 3′ telomeric tail, polyA tail or blunt end were generated by annealing 5′-GCAGTGTCTAGGTCGTGACTTT-3′ to 5′-GGAAAGTCACGACCTAGACACTGCGAGCTCGAATTCACTGGAGTGACCTC(TTAGGG)_4_-3′, 5′-GGAAAGTCACGACCTAGACACTGCGAGCTCGAATTCACTGGAGTGACCTC(A)_24_-3′, or 5′-GGAAAGTCACGACCTAGACACTGCGAGCTCGAATTCACTGGAGTGACCTC-3′, respectively; labeling the 3′ end with α-^32^P-dCTP; and annealing 5′-GAGGTCACTCCAGTGAATTCGAG-3′.

Plasmid substrate pTELN consists of 800 bp of telomere repeat cloned into the Xba1 and BamH1 sites of pBluescript (KS+), downstream of the T7 promoter [43]. Quikchange mutagenesis (Stratagene) generated an Nb.BbvC1 nickase site 131 bp upstream of the promoter and 101 bp from one of two PvuII sites. PvuII cleavage of intact 3.8 kb pTelN produces two fragments, 2.5 kb and 1.3 kb in length. Exonucleolytic digestion from the 5′-nick destroys the promoter-proximal PvuII site, so the fraction of 3.8 kb molecules produced upon PvuII digestion provides a measure of exonucleolytic activity [42].

### Enzymes and Enzyme Assays

hEXO1 was expressed from a baculovirus vector in sf9 insect cells and purified as described [42], and hFEN1 was obtained commercially (Trevigen). Activity assays were carried out in 20 µl containing indicated amounts of enzyme and 5 nM DNA substrate; in 30 mM HEPES, pH 7.6, 40 mM KCl, 8 mM MgCl_2_, 0.1 mg/ml BSA and 1 mM DTT for hEXO1, in manufacturer's buffer supplemented with 40 mM KCl to maintain stability of G4 structures. Products of digestion of labeled synthetic oligonucleotides were denatured by heating at 95°C for 10 min in 0.5 volume of 95% formamide/20 mM EDTA at pH 8.0, and a 5 µl aliquot resolved by denaturing gel electrophoresis on 8 M urea, 12% or 20% (for exonuclease assays) polyacrylamide gels. Products of digestion of plasmid substrates were resolved by native agarose gel electrophoresis and quantified by indirect end-labeling after transfer to a nylon membrane and hybridization to a labeled probe complementary to the T7 promoter [42]. Gels were scanned with a STORM Phosphorimager (Amersham) and label quantitated with Image Quant software (Amersham).

## Supporting Information

Figure S1 Structure formation by substrates. (A) Substrates diagrammed on left were analyzed by electrophoresis on a native gel containing 10 mM KCl. Substrates were 3′ end-labeled either opposite the tail or at the nick; asterisk denotes end-label. Retarded mobility on a native gel containing 10 mM KCl is diagnostic of G4 DNA formation by substrates b and e (arrow). (B) Substrates diagrammed on left (asterisk denotes 5′-end-label) were gel purified, and structure formation confirmed by electrophoresis on a native gel containing 10 mM KCl. Substrates bearing an unstructured 5′-flap (a) migrated more rapidly than substrates containing telomeric G4 DNA 5′ flap (b) or 3′ tail (c). These substrates (indicated by arrows) were used for subsequent assays.
(0.12 MB PDF)
