# Supplementary figures and images for: Distinct Activities of Exonuclease 1 and Flap Endonuclease 1 at Telomeric G4 DNA
Source: PLoS One. 2010 Jan 26;5(1):e8908. doi: 10.1371/journal.pone.0008908 (PMC2811187; doi:10.1371/journal.pone.0008908)

**Figure S1**

**A**

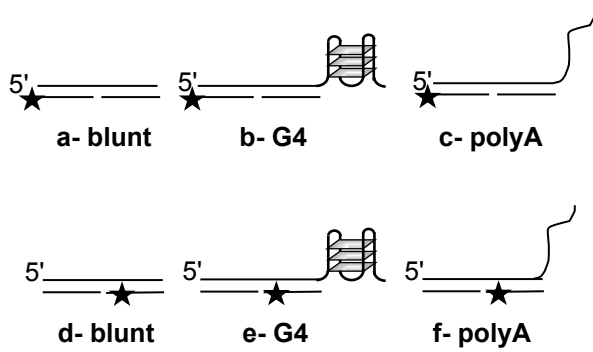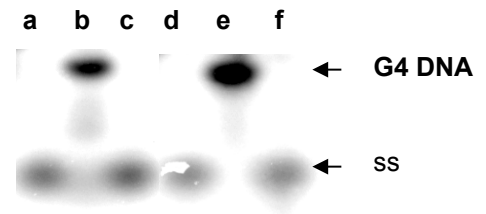

**B**

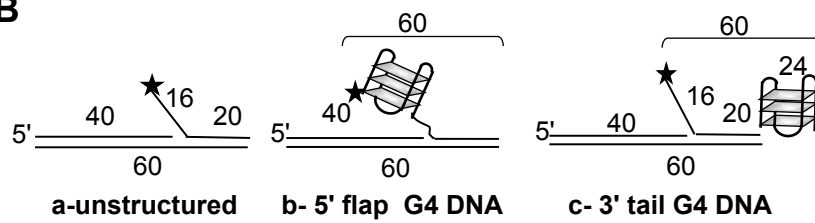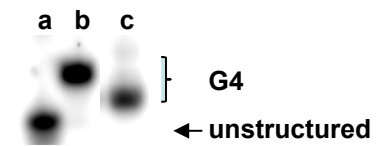

Supplement: Figure S1 — Structure formation by substrates. (A) Substrates diagrammed on left were analyzed by electrophoresis on a native gel containing 10 mM KCl. Substrates were 3′ end-labeled either opposite the tail or at the nick; asterisk denotes end-label. Retarded mobility on a native gel containing 10 mM KCl is diagnostic of G4 DNA formation by substrates b and e (arrow). (B) Substrates diagrammed on left (asterisk denotes 5′-end-label) were gel purified, and structure formation confirmed by electrophoresis on a native gel containing 10 mM KCl. Substrates bearing an unstructured 5′-flap (a) migrated more rapidly than substrates containing telomeric G4 DNA 5′ flap (b) or 3′ tail (c). These substrates (indicated by arrows) were used for subsequent assays. (0.12 MB PDF) [file pone.0008908.s001.pdf]
